# Comparisons of adipogenesis- and lipid metabolism-related gene expression levels in muscle, adipose tissue and liver from Wagyu-cross and Holstein steers

**DOI:** 10.1371/journal.pone.0247559

**Published:** 2021-02-24

**Authors:** Li Liu, Peili Cao, Lupei Zhang, Meiyu Qi, Liang Wang, Zhongqiu Li, Guang Shao, Liyan Ding, Xiuhua Zhao, Xiaochuan Zhao, Shanshan Xu, Haifeng Zhang, Jinbao Chai, Mengmeng Yue, Genlin Wang, Di Liu, Fang Sun

**Affiliations:** 1 Key Laboratory of Combining Farming and Animal Husbandry of Ministry of Agriculture, Institute of Animal Husbandry, Heilongjiang Academy of Agricultural Sciences, Harbin, China; 2 Heilongjiang Journal Press of Agricultural Science and Technology, Heilongjiang Academy of Agricultural Sciences, Harbin, China; 3 Institute of Animal Science, Chinese Academy of Agricultural Sciences, Beijing, China; 4 Branch of Animal Husbandry and Veterinary of Heilongjiang Academy of Agricultural Sciences, Qiqihar, China; 5 College of Animal Science and Veterinary Medicine, Heilongjiang Bayi Agricultural University, Daqing, China; Universidade Federal de Viçosa, BRAZIL

## Abstract

The intramuscular fat (IMF) content and fatty acid composition are important meat quality traits that are mostly affected by the cattle breed. Muscle, adipose tissue and liver are important organs involved in the development of intramuscular adipose tissue. Thus, we hypothesized that there were marked differences in the adipogenesis and lipid metabolism of these tissues between Wagyu-cross and Holstein steers during the finishing phases. To test this hypothesis, we analyzed the expression levels of adipogenesis- and lipid metabolism-related genes in longissimus muscle (LM), subcutaneous fat (SCF) and liver from Wagyu-cross and Holstein steers at 26 months of age. The IMF content and fatty acid profile of LM were determined. Wagyu-cross steers had a higher IMF content and MUFA percentages in the LM than Holstein steers (*P*<0.05). The relative expression of *FGF2*, *COL1A1*, *SREBP1c*, *SCD1*, *GRP78* and *LEP* was greater in the LM of Wagyu-cross steers than in Holstein steers (*P*<0.05). In contrast, Holstein steer SCF had higher (*P*<0.05) mRNA expression levels of *FABP4* and *ADIPOQ* than Wagyu-cross steers. In the liver, the expression of *SREBP1c* and *GRP78* in Wagyu-cross steers was significantly higher than that in Holstein steers (*P*<0.05). The results demonstrate that both intramuscular adipogenesis and fibrogenesis are enhanced in Wagyu-cross steers compared with Holstein steers during the finishing phase and that IMF deposition is positively correlated with the maturity of SCF and hepatic lipid accumulation in Wagyu-cross steers.

## Introduction

Marbling or intramuscular fat (IMF) contents are an important trait determining the quality of beef and directly affect the taste, juiciness, and tenderness of meat [1]. IMF can be enhanced due to both adipogenesis and lipid metabolism. The initial stage of adipogenesis mainly concerns the conversion from fibro/adipogenic progenitor cells to preadipocytes [2], which is regulated by multiple signals, including extracellular factors such as fibroblast growth factors (FGFs) [3], transforming growth factor beta (TGFβ) [4] and extracellular matrix (ECM) components [5]. Intracellular regulators mainly include zinc-finger protein 423 (ZNF423) [6]. Enhancing preadipocyte differentiation of progenitor cells increases the IMF content in cattle.

During the terminal stage of adipogenesis, there are also several key transcription factors that control the differentiation of preadipocytes into mature adipocytes, such as CAAT/enhancer binding proteins (C/EBPs) [7], peroxisome proliferator-activated receptor γ (PPARγ) [8, 9], and sterol regulatory element-binding protein-1c (SREBP1c) [10], which induce the expression of many downstream target genes involved in lipid metabolism.

Communication among multiple organ plays an important role in lipid metabolism, and there is a clear interaction between muscle, subcutaneous fat (SCF) and liver in ruminant lipid metabolism. The liver carries out central metabolic functions; for example, long-chain fatty acids (LCFAs) in the liver are esterified to produce triglycerides (TG), which are packaged into very-low-density lipoproteins (VLDL) and then exported to muscle and adipose tissues by the bloodstream [11]. SCF is not only a fuel reservoir that supplies nonesterified fatty acids (NEFAs) for muscle and liver [12] but also an endocrine organ that produces numerous bioactive factors such as adipokines that communicate with muscle and liver [13]. Therefore, it is necessary to monitor the lipid metabolism of SCF and the liver, which affect the development of IMF.

In addition to IMF, the intramuscular fatty acid composition also contributes importantly to meat quality [14], and beef rich in monounsaturated fatty acids (MUFAs) and polyunsaturated fatty acids (PUFAs) can decrease the risks of cardiovascular disease in humans [15]. In former studies, it was shown that the cattle breed could significantly affect the IMF content and fatty acid composition [16–21]. Japanese black cattle (Wagyu) have the unique ability to store enormous amounts of fat and MUFAs within the muscle, which is a good animal model to study adipogenesis and lipid metabolism [22]. In recent years, there has been growing demand for high-marbling beef in China, where a certain number of Wagyu-cross cattle have been farmed. In addition, Holstein steers, which have the genetic potential to deposit IMF with relatively little SCF, are starting to be raised for a high quality and profitable beef product in dominant milk-producing provinces in China. Based on the IMF difference, a series of comparative studies were carried out between Wagyu and Holstein steers, which revealed that Wagyu had higher terminal differentiation activity of adipocytes in the *longissimus* muscle (LM) and SCF than the Holstein steers [17, 23–27]. However, little information is available regarding the differences in the molecular regulation of preadipocyte determination and liver lipid metabolism between these two breeds in the finishing phase. Therefore, we hypothesized that there were marked differences in adipogenesis and lipid metabolism of LM, subcutaneous fat (SCF) and liver between Wagyu-cross and Holstein steers at 26 months of age.

## Material and methods

### Ethics statement

The research was undertaken with the approval of the Animal Ethics Committee of the Institute of Animal Husbandry, Heilongjiang Academy of Agricultural Sciences (HAAS) (Harbin, China). All procedures were in strict accordance with the guidelines proposed by the China Council on Animal Care.

### Animals and sample collection

Wagyu-cross (Snow dragon beef, the offspring of Wagyu and Filial 1 hybrid (F1) crossbred cows of Limousin by Fuzhou yellow cattle, n = 3) and Holstein steers (n = 3) were kept under the same conditions from 11 to 26 months of age. Steers of both breeds were raised individually in pens with the free stall feeding system. Each animal had 9 m^2^ for normal activities, and the pen was cleaned every day. All steers were fed twice daily a high-energy diet and given free access to water and mineral blocks. The diet compositions are shown in Table 1. Body weights were measured monthly, and feed intakes of the groups were recorded daily.

**Table 1:** Composition of the experimental diets.

Item	Early	Middle	Late
Ingredient (%)			
Flaked corn	63	57	50
Flaked barley		7	14
Soybean meal	17.5	12.5	13
Wheat bran	17.5	13.5	11
Salt	4	4	4
Concentrate:roughage^a^	82:20	85:15	88:12
Nutrient composition, % of DM			
Crude protein	12.5	11.5	10.5
Total digestible nutrients	76	79	83

^a^ Corn stalk or rice straw was used as roughage in all diets. Early, middle, and late diets for two groups of steers were used in the first phase from 11 to 15 months of age, in the second phase from 16 to 22 months of age and in the third phase from 23 to 26 months of age, respectively.

Immediately after slaughter, samples of LM, SCF (between the 12th and 13th ribs) and liver tissue were collected from each animal, minced, snap frozen in liquid nitrogen promptly and subsequently stored at -80°C. Additionally, a 2- to 3-cm-thick muscle slice was removed from the 12th rib area of the LM, fixed in 4% paraformaldehyde and embedded in paraffin. A second sample of LM was vacuum-packed and stored at -80°C until determination of intramuscular fat content and fatty acid composition.

### Postslaughter measurements

Carcass, liver and perirenal fat weights were recorded after slaughter. The LMs were chopped and lyophilized using a freeze-drying machine, and samples were analyzed in triplicate for crude fat (method 960.39; AOAC, 2000).

### Fatty acid analysis

Total lipids were extracted from LM via chloroform/methanol (2:1, v/v) extraction, and fatty acid methyl ester (FAME) synthesis was conducted according to the methods described by Nuernberg et al. (2010). FAMEs were separated by gas chromatography (6890N; Agilent Technologies, Santa Clara, California) using a capillary column (Supelco SP. 2560; 0.25 mm by 0.20 μm by 100 m; Sigma-Aldrich Chemie GmbH, Munich, Germany) and a flame ionization detector. For the separation of FAMEs from samples, the following temperature program was applied with nitrogen as the carrier gas at a flow rate of 1 ml/min: from 150 to 165°C at 1°C/min, then increased to 167°C at 0.2°C/min, from 167°C to 225°C at 1.5°C/min, and the temperature was maintained for 15 min. The injector and detector were maintained at 250°C. Individual fatty acids were identified by comparison with retention times of standards, which were purchased from the Matreya Company (State College, Pennsylvania) and Sigma-Aldrich Canada.

### Histology and image analysis

Five-micrometer sections were prepared from LM tissue blocks from each animal, and the sections were deparaffinized, rehydrated and stained with hematoxylin-eosin (H/E). Intramuscular adipocyte and muscle fiber size were estimated by measuring the average diameter of at least 200 cells using the interpolating polygon function of ImageJ software (National Institute of Health, USA), and the density of muscle fibers (fibers/mm^2^) was estimated by the point tool using 300 points. To evaluate the percentage of the endomysium area, a microscopic image of H/E-stained muscle fibers was transformed into a grayscale 8-bit image, which was further binarized into muscle fibers (black), perimysium (white) and endomysium (white). Then, the perimysium area was blackened with a brush tool; thus, the percentage of endomysium area was detected using a threshold operation.

### RNA extraction and quantitative real-time PCR

Total RNA was extracted from LM, SCF and liver samples using TRIzol reagent (Invitrogen, Carlsbad, CA, USA), and approximately 1 μg of RNA from each sample was reverse transcribed into cDNA using the PrimeScript^™^ RT reagent Kit with gDNA Eraser (TaKaRa). Real-time PCR was performed using SYBR^®^ Premix Ex Taq^™^ II (Tli RNaseH Plus) (TaKaRa). All data were normalized by RPLP0 as an internal control. Relative expression levels of each gene between the two cattle breeds were calculated using the 2^-ΔCt^ method [28], where ΔCt = Ct(target genes) − Ct(RPLP0). The fold-change value was calculated using the formula: mean 2^-ΔCT^ (high)/mean 2^-ΔCT^ (low). Primers are listed in Table 2.

**Table 2:** List of primers.

Gene name	Abbreviation	Primer sequence (5’→3’)		Accession	Product size
Fibroblast growth factor 2	FGF2	F	GTGCAAACCGTTACCTTGCTAT	NM_174056.4	157bp
		R	GTTCGTTTCAGTGCCACATACC		
Fibroblast growth factor receptor 1	FGFR1	F	ATGGACTCCGTGGTGCCTTCG	NM_001110207.1	244bp
		R	TCCCGTTCACCTCAATGTGCTTC		
Transforming growth factor beta 1	TGFβ1	F	CAATTCCTGGCGCTACCTCA	NM_001166068.1	127bp
		R	GCGAAAGCCCTCTATTTCCTCT		
Collagen, type I, alpha 1	COL1A1	F	CCACCCCAGCCGCAAAGAGT	NM_001034039	163bp
		R	ACGCAGGTGACTGGTGGGATGTC		
Zinc finger protein 423	ZNF423	F	GAAGGCATCAACCATGAGTGTAAG	NM_001101893.1	141bp
		R	CTGGACGAAGACTGTGAAGCAC		
Sterol regulatory element-binding protein1c	SREBP1c	F	CCGAGGCCAAGTTGAATAAATCT	NM_001113302.1	147bp
		R	ACACCAGGTCCTTCAGCGATTTG		
CCAAT enhancer binding protein alpha	CEBPα	F	GCAAAGCCAAGAAGTCCG	NM_176784.2	183bp
		R	GGCTCAGTTGTTCCACCCGCTT		
Peroxisome proliferator activated receptor gamma 2	PPARγ2	F	TCTGCAAGGACCTCACAAGA	NM_181024.2	173bp
		R	TCATAGTGCGGAGTGGAAAT		
Lipoprotein lipase	LPL	F	ACGATTATTGCTCAGCATGG	NM_001075120.1	130bp
		R	ACTTTGTACAGGCACAACCG		
Adipocyte differentiation related protein	ADRP	F	GCCGAGTTACTATGTTAGACT	NM_173980.2	219bp
		R	AGCCAGGACAGATAGAGC		
Fatty acid binding protein 4	FABP4	F	TGGAAACTTGTCTCCAGTGAAA	NM_174314.2	120bp
		R	ACCCCCATTCAAACTGATGA		
Acetyl-CoA carboxylase alpha	ACACA	F	GAGACAAACAGGGACCATT	NM_174224.2	145bp
		R	ATCAGGGACTGCCGAAAC		
Stearoyl-CoA desaturase 1	SCD1	F	GTGATGTTCCAGAGGAGGTACTACAA	NM_173959.4	95bp
		R	AACGTTTCATCCCACAGATACCA		
Glucose-regulated protein 78	GRP78	F	AACGACCCCTGACGAAAGAC	NM_001075148.1	129bp
		R	TCACTCGAAGAATGCCATTCAC		
Leptin	LEP	F	CGATTCCTGTGGCTTTGG	NM_173928.2	178bp
		R	GGAGCCCAGGGATGAAGT		
Adiponectin, C1Q and collagen domain containing	ADIPOQ	F	ATCGCCTCCTACTTCCACCCTG	NM_174742.2	130bp
		R	TTGTCCTCGCCATGACTGGGT		
Ribosomal protein lateral stalk subunit P0	RPLP0^a^	F	TTCTCCTTCGGGCTGGTCAT	NM_001012682.1	167bp
		R	GGTACAGATGCGACGGTTGG		

^a^ Housekeeping gene.

### Statistical analyses

Statistical analysis was performed using SAS statistical software (SAS Institute, Cary, NC, USA). The phenotypic data, histological analysis data and mRNA abundance between the two breeds were evaluated by Student’s t-test. All results are presented as the mean ± SE, and statistical significance was considered at *P*<0.05. Pearson correlation coefficients among differentially expressed genes (DEGs), as well as correlation coefficients between DEGs and the IMF content or fatty acid composition in the LM, were calculated using the CORR procedure of SAS. Cytoscape software was utilized to construct the gene association network based on the Pearson correlation coefficient among DEGs in the LM, with a *p-*value<0.01 as the threshold. Important nodes were reflected by computing the betweenness centrality (BC). High BC nodes are highlighted in large circles and red color. All the nodes were placed in hierarchically arranged layers using yFiles layouts in Cytoscape. A heatmap was generated to visualize the correlation between the DEGs and fatty acid composition in the LM. A principal component analysis (PCA) was performed using the relative gene expression data of three tissues in originPro 2018.

## Results

### Phenotypic data

The phenotypic data for Wagyu-cross and Holstein steers at 26 months of age are presented in Table 3. The Holstein steers had significantly higher initial body weight, slaughter weight, average daily gain and HCW than the Wagyu-cross steers (*P<*0.01), and the perirenal fat percentage tended to be greater in Holstein steers (*P* = 0.08). Conversely, the IMF content was higher in Wagyu-cross steers (*P* = 0.03).

**Table 3:** Phenotypic data of Wagyu-cross and Holstein steers at 26 months of age (LSMEAN ± SE).

Trait	Wagyu-cross	Holstein	*P*-value
Initial body weight (11months of age), kg	348.6±12.38	446.6±21.39	<0.01
Slaughter weight (SW), kg	683.0±17.35	938.3±21.62	<0.01
Average daily gain, g/day	713.0±15.63	1048.47±61.19	<0.01
Hot carcass weight (HCW), kg	393.1±23.22	538.3±28.53	<0.01
Intramuscular fat content, %	15.72±0.53	11.86±1.01	0.03
Perirenal fat weight, % HCW	2.3±0.26	3.0±0.21	0.08
Liver weight, % SW	0.82±0.05	0.83±0.04	0.41

### Fatty acid composition of LM

The fatty acid composition of LM in Wagyu-cross and Holstein steers is presented in Table 4. Compared with Holstein steers, the percentages of C14:1 (*P* = 0.01), C16:1 (*P* = 0.05) and MUFAs (*P* = 0.04) were higher in Wagyu-cross steers, whereas the percentages of C18:0 (*P* = 0.02) and C20:3n6 (*P* = 0.04) were lower in Wagyu-cross steers, and the percentages of C20:4n6 (*P* = 0.06), SFAs (*P* = 0.09), and PUFAs (*P* = 0.07) tended to be lower in Wagyu-cross steers.

**Table 4:** Fatty acid composition (% of total fatty acids) of LM in Wagyu-cross and Holstein steers.

Breed	Wagyu-cross	Holstein	*P*-value
C14:0	3.62±0.16	3.18±0.37	0.16
C14:1	1.26±0.14	0.81±0.07	0.01
C16:0	28.38±0.97	28.87±0.76	0.35
C16:1	5.70±0.51	4.63±0.22	0.05
C18:0	11.16±0.65	13.08±0.43	0.02
C18:1n9c	44.85±0.53	43.07±1.19	0.11
C18:2n6c	2.63±0.19	3.49±0.56	0.10
C18:3n3	0.21±0.01	0.23±0.02	0.15
C20:3n6	0.13±0.01	0.19±0.02	0.02
C20:4n6	0.21±0.02	0.43±0.12	0.06
∑SFA	44.21±1.15	46.37±0.84	0.09
∑MUFA	52.62±1.2	49.3±0.97	0.04
∑PUFA	3.17±0.22	4.33±0.66	0.07

∑SFA, saturated fatty acid, without any double bonds (C14:0, C15:0, C16:0, C17:0 and C18:0); ∑MUFA, monounsaturated fatty acid, with a single double bond (C14:1 C16:1, C17:1 and C18:1); ∑PUFA, polyunsaturated fatty acid, all fatty acids with two or more double bonds (including C18:2n6c, C18:3n3, C20:3n6 and C20:4n6).

### Muscle structure trait

**Fig 1:**
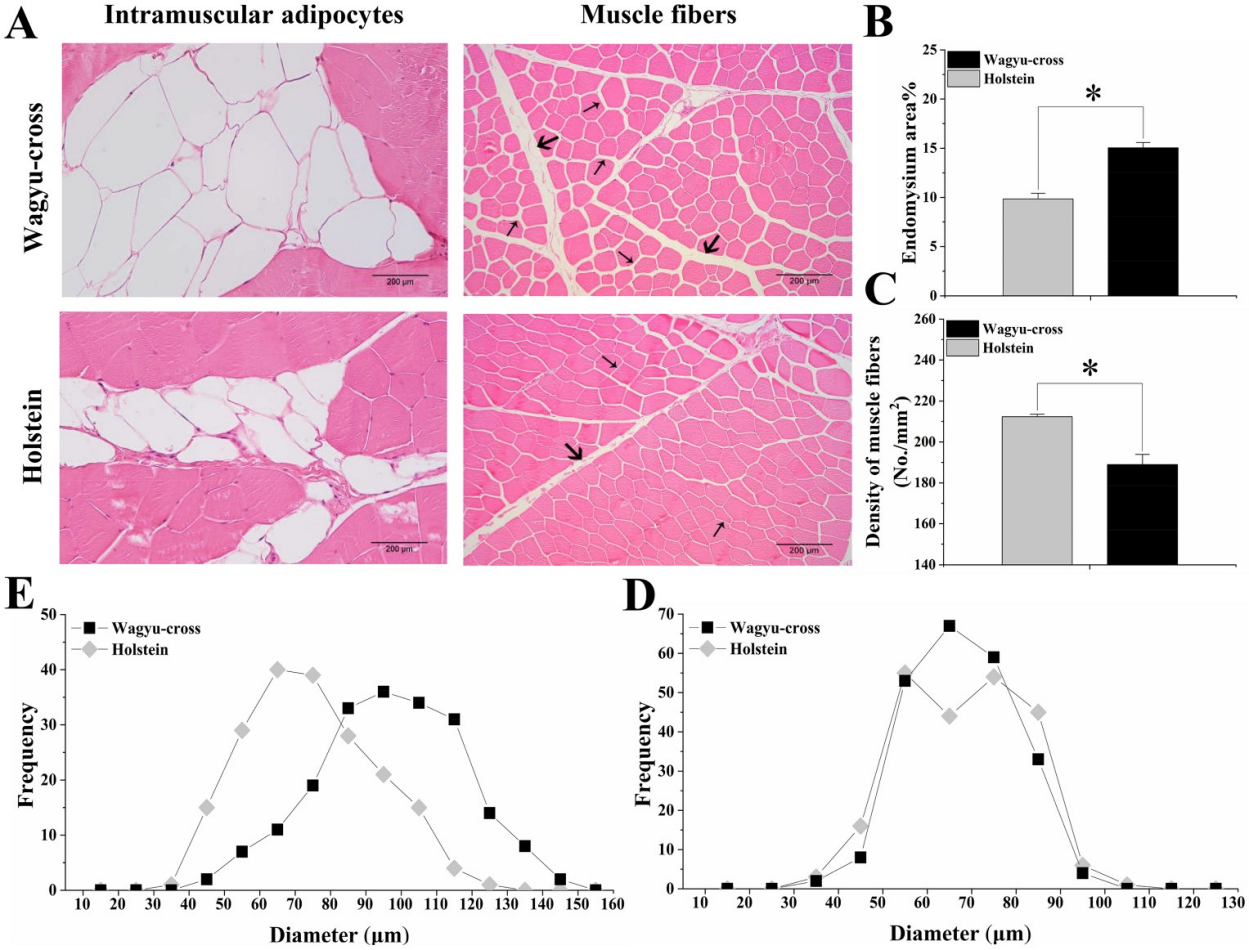
Histological analysis of muscle cross-sections in Wagyu-cross and Holstein steers. (A) HE staining of intramuscular adipocytes and muscle fibers. Scale bar: 200 μm. Large arrows indicate perimysium, and small arrows indicate endomysium. (B) Endomysium area %. (C) Density of muscle fibers. (D) Frequency distribution of muscle fiber diameter. (E) Frequency distribution of intramuscular adipocyte diameter.

As shown by HE staining (Fig 1A), the endomysium between the muscle fibers occupied more space in the Wagyu-cross steers, which had a higher percentage of endomysium area than the Holstein steers (*P*<0.05) (Fig 1B). The density of muscle fibers was higher in Holstein steers (*P*<0.05) (Fig 1C). There were no differences in muscle fiber size between the two cattle breeds (Fig 1D), whereas the size distribution of intramuscular adipocytes showed that the Wagyu-cross steers had larger adipocytes than Holstein steers (Fig 1E).

### Gene expression in LM

**Fig 2:**
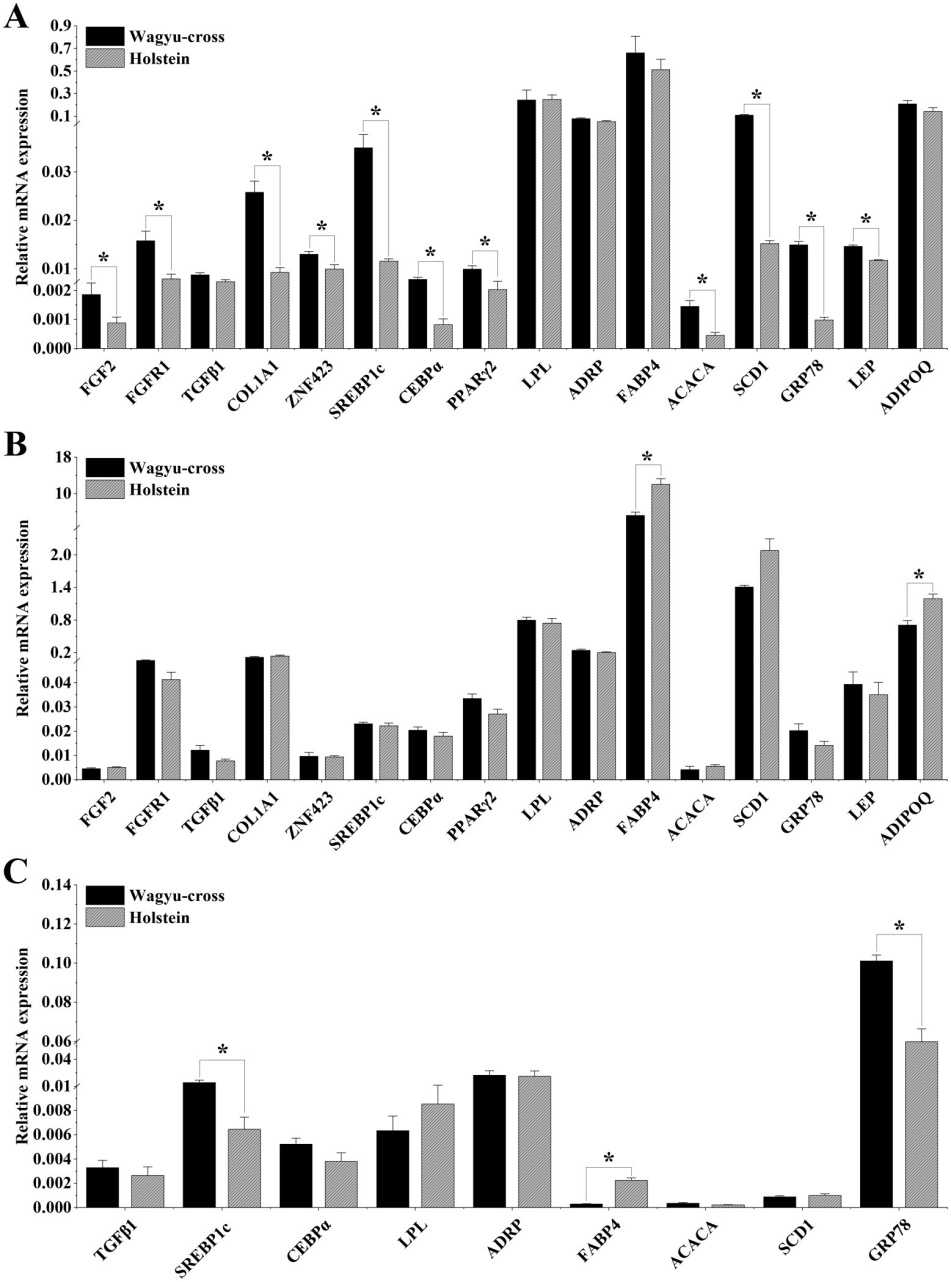
Relative gene expression in the LM (A), SCF (B) and liver (C) from Wagyu-cross and Holstein steers. The expression levels were calculated using the 2^-ΔCt^ method and were normalized to RPLP0. Bar values represent the mean ± SEM, and asterisks indicate *p*<0.05 between the two groups.

The relative mRNA expression levels of the sixteen adipogenesis- and lipid metabolism-related genes in the LM of the two cattle breeds are presented in Fig 2A. *FGF2*, *FGFR1*, *COL1A1*, *ZNF423*, *SREBP1c*, *CEBPα*, *PPARγ2*, *ACACA*, *SCD1*, *GRP78* and *LEP* in the LM showed higher expression levels in Wagyu-cross steers when compared with Holstein steers (*P<*0.05), corresponding to a fold-change of 2.11, 3.77, 4.93, 1.63, 4.60, 3.42, 2.42, 3.19, 10.82, 10.13 and 1.43, respectively. *TGFβ1* tended to have higher expression levels (1.61-fold) in Wagyu-cross steers than in Holstein steers (*P* = 0.078). However, no significant differences were found in the mRNA expression of *LPL*, *ADRP*, *FABP4* and *ADIPOQ* between the two cattle breeds.

### Gene expression in SCF

The relative mRNA expression levels of the sixteen adipogenesis- and lipid metabolism-related genes in the SCF of the two cattle breeds are presented in Fig 2B. *FABP4* and *ADIPOQ* in SCF showed higher expression levels in Holstein steers than in Wagyu-cross steers (*P<*0.05), corresponding to fold changes of 2.31 and 1.69, respectively. *SCD1* tended to have higher expression levels (1.47-fold) in Holstein steers than in Wagyu-cross steers (*P* = 0.09).

### Gene expression in liver

The relative mRNA expression levels of the nine adipogenesis- and lipid metabolism-related genes in the livers of the two cattle breeds are presented in Fig 2C. Significant differences were found in the gene expression levels of *SREBP1c*, *FABP4* and *GRP78* between Wagyu-cross and Holstein steers, and *SREBP1c* and *GRP78* had higher expression levels (2.19-fold and 1.7-fold) in Wagyu-cross than in Holstein steers. Conversely, the *FABP4* gene showed higher expression levels (7.62-fold) in Holstein steers than in Wagyu-cross steers. *ACACA* tended to have higher expression levels (1.64-fold) in Wagyu-cross steers than in Holstein steers (*P* = 0.096). In addition, *ACACA*, *SCD1* and *FABP4* were expressed at low levels in the liver.

### Relationships among the expression levels of genes in LM

**Fig 3:**
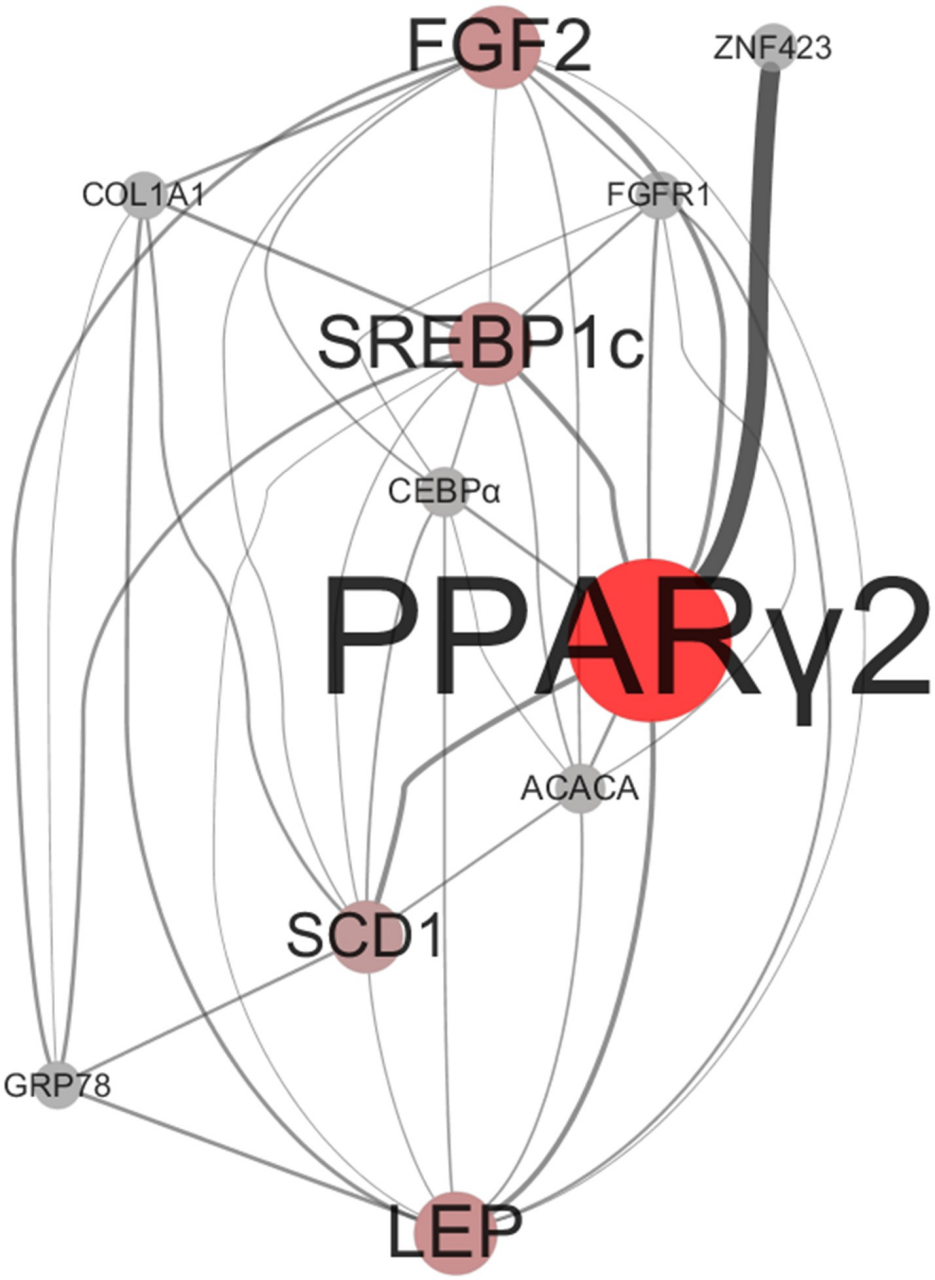
Gene association network composed of five hub genes. Node color and size: red and larger nodes represent hub genes with more links. *PPARγ2* appears to be the super hub gene in the network with the highest connectivity.

To display the correlation between DEGs in the LM more clearly, Cytoscape software was utilized to construct a gene association network, which contained 11 nodes and 37 edges. Each node of the Cytoscape network represents a gene, and the edge between nodes represents a possible regulatory relationship between genes. As shown in Fig 3, adipogenesis- and lipid metabolism-related genes were placed in hierarchically arranged layers from top to bottom. Based on the betweenness centrality (BC) values of each node in the network, *FGF2*, *SREBP1c*, *PPARγ2*, *SCD1* and *LEP* were identified as hub nodes, displaying higher connectivity within the network. *PPARγ2* had the highest BC, occupying the center of the network. *FGF2* was at the top of the network and was significantly positively correlated with most genes except *ZNF423*, which was only significantly correlated with *PPARγ2*. In addition, the expression of *FGFR1* was positively correlated with that of *SREBP1c*, *CEBPα*, *PPARγ2*, *ACACA* and *LEP*. There was a significant positive correlation among *FGF2*, *COL1A1*, *SREBP1c*, *GRP78*, *SCD1* and *LEP*.

### Relationships among gene expression levels, IMF content and fatty acid composition in LM

**Fig 4:**
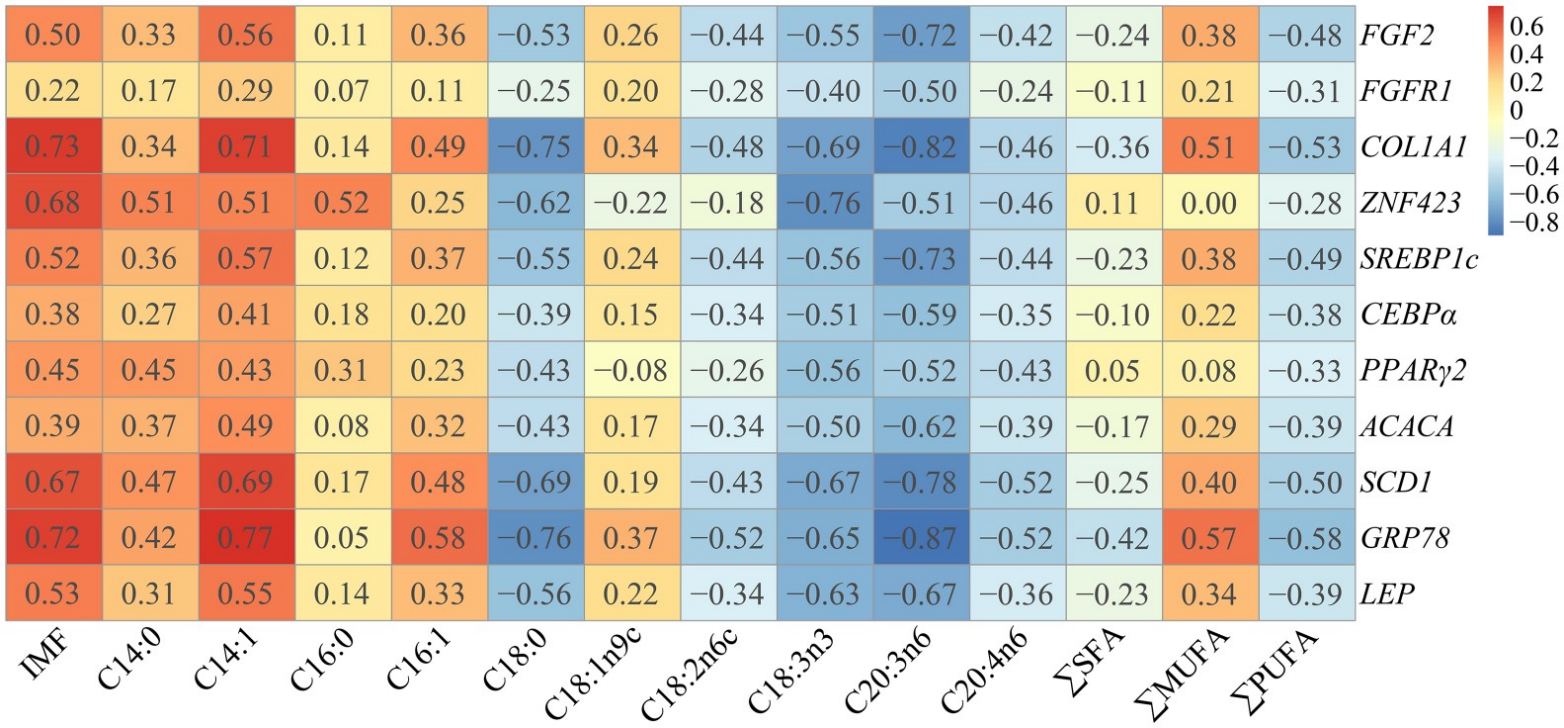
Correlations among gene expression levels, IMF content and fatty acid composition in the LM. The number in each cell represents the correlation coefficient.

The correlation among DEGs in the LM, IMF content and fatty acid percentages is depicted in Fig 4. It is worth noting that *COL1A1*, *SCD1* and *GRP78* gene expression showed a strong positive association with IMF content, the percentage of C14:1 and moderate positive correlations with the percentages of C16:1 and MUFAs. Most gene expression showed a negative association with the percentages of C18:0, SFAs, C18:1n9c, C18:3n3, C20:3n6, C20:4n6 and PUFAs.

### PCA of the gene expression in LM, SCF and liver

**Fig 5:**
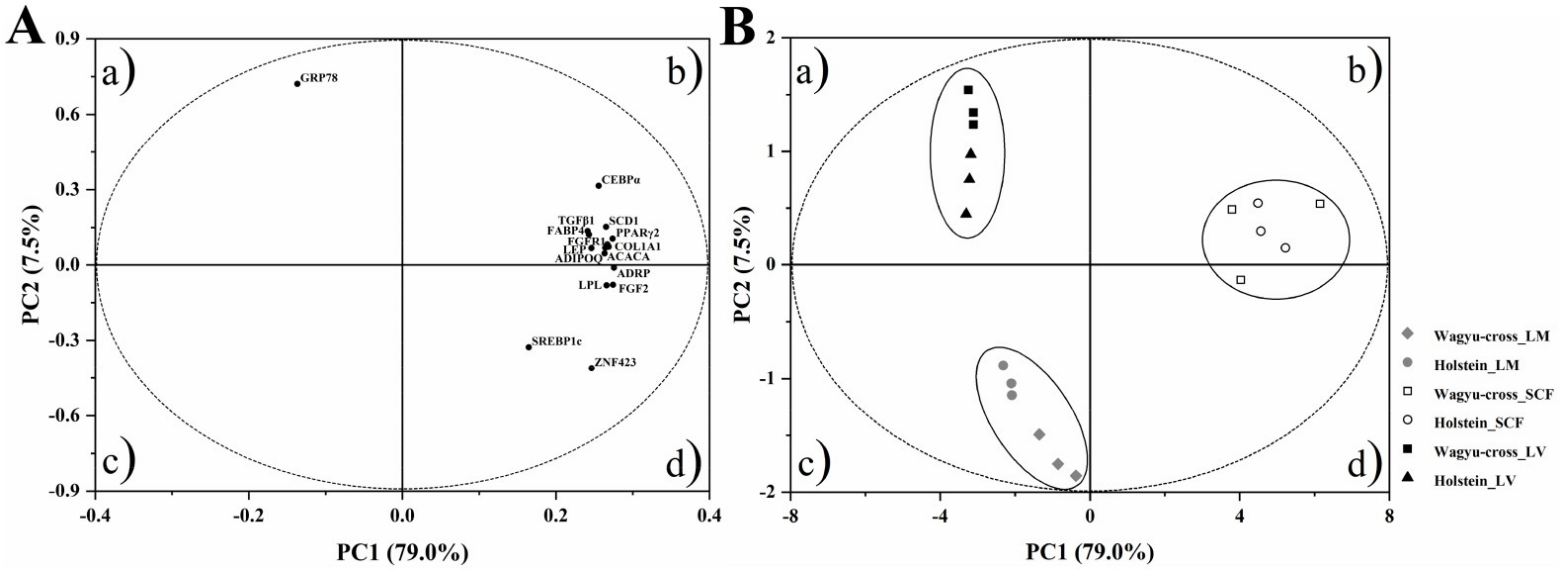
Loading plot (A) and score plot (B) of the principal component analysis (PCA) for gene expression in the LM, SCF and liver. LM: *longissimus* muscle, SCF: subcutaneous fat, LV: liver.

A principal component analysis (PCA) of the gene expression of liver, LM and SCF was performed to highlight the differences in adipogenesis and lipid metabolism among the three tissues and between the two breeds. The first two principal components together explained 86.5% of the total variance, with 79.0% for PC1 and 7.5% for PC2. In the loading plot, most genes were allocated to quadrant *b*, which was responsible for forming the SCF cluster (Fig 5A). In quadrant *a*, the liver cluster was only defined by the *GRP78* gene. In addition, *SREBP1c* and *ZNF423* were separated from the other variables in quadrant *d*. The score plot showed that eighteen tissue samples were clearly divided into three clusters, corresponding to each tissue (Fig 5B). The tissue samples of the two breeds were clearly separated in each cluster except SCF in which Wagyu-cross steers samples were scattered. The SCF cluster was separated from the liver and LM clusters along PC1, while PC2 separated the liver cluster from the LM cluster.

## Discussion

Recently, some researchers showed that the IMF content and intramuscular fatty acid composition depended not only on adipogenesis but also on lipid metabolism in the muscle, adipose tissue and liver of cattle [29–31]. In the present study, we showed that Wagyu-cross steers had higher IMF content and percentages of C14:1, C16:1 and MUFAs in the LM than Holstein steers at 26 months of age. To elucidate the molecular mechanisms involved in these physiological differences. A comparative analysis was performed to investigate the expression levels of adipogenesis- and lipid metabolism-related genes in the LM, subcutaneous fat (SCF) and liver from Wagyu-cross and Holstein steers. As a result, we observed greater mRNA expression of *SREBP1c*, *CEBPα*, *PPARγ2*, *ACACA* and *SCD1* in the LM of Wagyu-cross steers than in that of Holstein steers. *SREBP1c*, *CEBPα* and *PPARγ2* are key transcription factors involved in adipogenesis and fat formation in vitro and in vivo [10, 32, 33] that promote the expression of *ACACA* and *SCD1*. Acetyl-CoA carboxylase alpha (ACACA) is a key rate-limiting enzyme of de novo fatty acid biosynthesis, and it has been reported that there is a positive relationship between ACACA enzyme activity and the IMF content in cattle [34], which is consistent with our findings. Stearoyl-CoA desaturase (SCD) is an endoplasmic reticulum (ER) enzyme that converts SFAs into MUFAs in mammalian adipocytes [35]. In the present study, the expression levels of *SCD1* were higher in the LM of Wagyu-cross steers than in Holstein steers, consistent with the finding that Wagyu-cross steers had higher percentages of C14:1, C16:1 and MUFAs in the LM than Holstein steers. In addition, we detected that Wagyu-cross steers had a higher expression level of glucose-regulated protein 78 (GRP78) in the LM, which is a marker gene of ER stress and can repair misfolded proteins. We speculated that higher IMF deposition might induce muscle damage and ER stress in the LM of Wagyu-cross steers [36]. Muscle histological evidence showed that the muscle fibers were looser in the LM of Wagyu-cross steers than in the Holstein steers, indicating that the structure of the endomysium was broken in the LM of Wagyu-cross steers. Therefore, the upregulation of *GRP78* might play an important role in muscle repair [37]. On the other hand, higher IMF deposition caused a higher expression level of the *LEP* gene in the LM of Wagyu-cross steers. Leptin (LEP) is a negative feedback signal in the regulation of lipid metabolism balance that inhibits lipid accumulation and promotes fatty acid oxidation [38]. A previous study revealed a positive correlation between the mRNA level of the *LEP* gene and adipocyte size in crossbred steers [39], which is consistent with the results of histological analysis.

Adipogenesis mainly consists of two stages: adipocyte determination and differentiation. Although adipocyte determination mainly occurs in the early stage of cattle development, higher mRNA expression of some key factors responsible for commitment to adipogenesis was still detected in the LM of Wagyu-cross steers at 26 months of age. Zinc-finger protein 423 (ZNF423) was identified as a transcriptional regulator of preadipocyte determination and controls *PPARγ* expression [6]. In this study, we observed greater mRNA expression of *ZNF423* in the LM of Wagyu-cross steers than in Holstein steers, indicating that there are differences in early adipogenesis in the LM from both breeds at 26 months of age. To further investigate the preadipocyte developmental potential in the LM of Wagyu-cross steers, we detected the expression of fibroblast growth factor 2 (FGF2) and its receptor fibroblast growth factor receptor 1 (FGFR1), which are known to regulate adipose-derived mesenchymal stem cell proliferation [40, 41]. Our results showed that *FGF2* and *FGFR1* expression was greater in the LM of Wagyu-cross steers than in that of Holstein steers. This result suggested that there were more abundant adipogenic progenitor cells in the LM of Wagyu-cross steers, which may be a key factor that contributed to the greater IMF content in the LM of Wagyu-cross steers compared with Holstein steers.

Because intramuscular adipocytes and fibroblasts originate from common progenitor cells named fibro/adipogenic progenitors (FAPs) [2], adipogenesis is inseparable from fibrogenesis, together forming adipose tissue and the extracellular matrix (ECM). Collagen type I is a major component of the ECM and is elevated by the TGF-β signaling pathway [42]. Recent studies have shown that TGF-β stimulates collagen, type I, alpha 1 (COL1A1) mRNA expression largely via autocrine expression of *FGF2* [43], which may explain why *COL1A1* expression was greater in the LM of Wagyu-cross steers than in Holstein steers, although transforming growth factor beta 1 (TGFβ1) tended to have higher expression levels in Wagyu-cross steers. Previous studies have shown that both adipogenesis and fibrogenesis in muscle were enhanced in Wagyu cattle compared with Angus cattle [16]. Therefore, our results again highlight the effect of breed on the control of adipogenesis and fibrogenesis in muscle.

We used a network-based approach to display the correlation (*P*<0.01) between DEGs in the LM of two cattle breeds. Surprisingly, DEGs were placed in hierarchically arranged layers from top to bottom, which was consistent with the molecular event of each stage in adipogenesis [44]. In this manner, *FGF2*, *SREBP1c*, *PPARγ2*, *SCD1* and *LEP* were identified as hub genes that play a critical role in adipogenesis and lipid metabolism. PPARγ2 formed the central molecule, showing the highest connectivity. In fact, it has been demonstrated that PPARγ2 is not only a key transcriptional regulator of adipogenesis but also a pivotal coordinator of the adipocyte differentiation process. PPARγ2 acts as an essential link between the regulator of early adipose commitment and the expression of mature adipocyte genes. Cells deficient in PPARγ2 are not capable of differentiating into adipocytes [45]. In addition, we found a possible interaction between *COL1A1* and *GRP78* in the network. This speculation is consistent with a previous report that depletion of *GRP78* decreased TGFβ1-induced *COL1A1* expression [46].

In contrast to what was observed in the LM, higher transcriptional activity of *FABP4* and *ADIPOQ* was detected in the SCF of Holstein steers compared with Wagyu-cross steers, suggesting that there were more active adipocytes in the SCF of Holstein steers. In addition, *SCD1* tended to have higher expression levels in the SCF of Holstein steers, which might contribute to lipid storage and to membrane biogenesis for adipocyte proliferation and differentiation [47, 48]. The expression of fatty acid binding protein 4 (*FABP4*) and adiponectin (*ADIPOQ*) is highly regulated during adipocyte differentiation and negatively correlated with obesity [49–51]. Together, our data show that the maturity status of adipocytes in Wagyu-cross steers was more advanced than that in Holstein steers at 26 months of age, which is consistent with a previous report [17].

The liver, as a central metabolic organ, plays an important role in fatty acid deposition and lipid metabolism. A previous study showed that the liver lipid metabolism of beef cattle was influenced by breed [31]. Our results showed greater mRNA expression of *SREBP1c* and *GRP78* in the liver of Wagyu-cross steers than in Holstein steer livers, which was consistent with what was observed in the LM. This result suggested that ER stress was induced in the liver of Wagyu-cross steers, which may be attributed to excessive hepatic SFA and cholesterol accumulation [52]. It has been reported that upregulated expression of *GRP78* prevents ER stress, promoting hepatic *SREBP1c* activation and reducing hepatic steatosis [53]. The liver is a primary target organ of adiponectin, which decreases hepatic lipogenesis by suppressing *SREBP1c* expression [54]. The low expression of *SREBP1c*, ACACA and *GRP78* in the liver of Holstein steers may be related to the high expression of adiponectin in adipose tissue [55]. The higher expression of *FABP4* in the liver of Holstein steers may be associated with inflammation [56].

The PCA showed the contrasting features among the tissues and breeds regarding adipogenesis- and lipid metabolism-related gene expression, showing a clear separation among the three tissues or between the two breeds. The statistical approach showed that most of the variables were responsible for fat cluster formation, suggesting that these genes are highly expressed in adipose tissue. In contrast, *GRP78* is the most important variable that kept liver samples in the first quadrant and is highly expressed in the liver. *SREBP1c* and *ZNF423*, which separated from the other variables, were primarily affected by the high expression of both genes in the LM.

## Conclusions

In summary, our findings demonstrated the impact of breed on the mRNA expression of adipogenesis- and lipid metabolism-related genes in the LM, SCF and liver. At 26 months of age, both adipogenesis and fibrogenesis in the LM were enhanced in Wagyu-cross steers compared with Holstein steers. Wagyu-cross steers had more advanced mature adipocytes in the SCF. In addition, we found that the expression of *GRP78* was higher in the liver of Wagyu-cross steers, likely indicating excessive hepatic lipid accumulation in Wagyu-cross steers compared with Holstein steers. These data support the notion that IMF deposition is positively correlated with the maturity of SCF and hepatic lipid accumulation in Wagyu-cross steers. The early maturity of the Wagyu-cross steers is more favorable for IMF deposition.
